# An effectiveness and economic analyses of tricalcium phosphate combined with iliac bone graft versus RhBMP-2 in single-level XLIF surgery in Thailand

**DOI:** 10.1186/s12891-023-06590-9

**Published:** 2023-06-19

**Authors:** Win Boonsirikamchai, Pochamana Phisalprapa, Chayanis Kositamongkol, Ekkapoj Korwutthikulrangsri, Monchai Ruangchainikom, Werasak Sutipornpalangkul

**Affiliations:** 1 Division of Orthopaedics, Bhumibol Adulyadej Hospital, Bangkok, Thailand; 2 Division of Ambulatory Medicine, Department of Medicine, Faculty of Medicine Siriraj Hospital, Mahidol University, Bangkok, Thailand; 3 Department of Orthopaedic Surgery, Faculty of Medicine Siriraj Hospital, Mahidol University, Bangkok, Thailand

**Keywords:** Bone morphologic protein, Tricalcium phosphate, XLIF, Economic analysis

## Abstract

**Study design:**

Retrospective study.

**Objectives:**

To perform effectiveness and economic analyses using data from a retrospective study of patients who underwent XLIF surgery using tricalcium phosphate combined with iliac bone graft (TCP + IBG) or BMP-2 in Thailand.

**Methods:**

Data were collected from retrospective review of the medical charts and the spine registry of Siriraj Hospital, Bangkok, Thailand. The patients were divided into two groups (TCP + IBG group and BMP-2 group). Demographic, perioperative data, radiographic, clinical results, and quality of life related to health were collected and analyzed at 2-year follow-up. All economic data were collected during the perioperative period and presented as total charge, bone graft, implant/instrumentation, operative service, surgical supply, transfusion, medication, anesthesia, laboratory, and physical therapy.

**Results:**

Twenty-five TCP + IBG and 30 BMP-2 patients with spondylolisthesis and spinal stenosis as primary diagnosis were included. There were no significant differences in all demographic parameters (gender, age, underlying disease, diagnosis, and level of spine) between these two groups. During the perioperative period, the TCP + IBG group had more mean blood loss and more postoperative complications compared to the BMP-2 group. At 2 years of follow-up, there were no significant differences between the radiographic and clinical outcomes of the TCP + IBG and BMP-2 groups. The fusion rate for TCP + IBG and BMP-2 at 2 years of follow-up was 80% and 96.7%, respectively, and no statistically significant differences were observed. All clinical outcomes (Utility, Oswestry Disability Index, and EuroQol Visual Analog Scale) at 2-year follow-up improved significantly compared to preoperative outcomes, but there were no significant differences between the TCP + IBG and BMP-2 groups, either at preoperatively or at 2-year follow-up. The total charge of TCP + IBG was statistically significantly lower than that of BMP-2. Furthermore, the charges of TCP + IBG and BMP-2 during the perioperative period in Thailand were up to three times less than those in the United States.

**Conclusions:**

Using TCP + IBG as a standalone bone substitution for XLIF surgery with additional posterior instrumentation resulted in significantly lower direct medical charge compared to those using BMP-2 in the perioperative period. However, we could not detect a difference in the long-term radiographic and clinical outcomes of patients with TCP + IBG and BMP-2. These suggest that TCP + IBG may be a valuable alterative bone graft, especially in low- and middle-income countries.

## Introduction

The prevalence of low back pain in Thailand generally ranges between 30% and 50% in the general population, with a tendency to increase due to the agricultural country and the aging society [1, 2]. Spinal fusion is a surgical treatment for degenerative lumbar spine diseases such as degenerative disc disease, spondylolisthesis, and spondylosis [3]. Due to the benefit of minimally invasive lateral lumbar spine fusion (XLIF) in terms of less blood loss, less surgical wound pain, rapid recovery time, and shorter return to work time, this procedure is popular for the treatment of degenerative lumbar spine in Thailand [4–7].

In the 1960s, Marshall Urist discovered Bone Morphogenetic Proteins (BMPs) which are part of the TGF-β superfamily. BMP-2 was isolated and produced using a recombinant technique (recombinant human bone morphogenetic protein-2, rhBMP-2) [8, 9]. Several in vitro and in vivo studies have demonstrated that rhBMP-2 has significantly higher fusion rates compared to autogenous bone graft [7].

Tricalcium phosphate-based ceramics (TCP) are widely accepted as biocompatible osteoconductive materials with chemical and structural properties that mimic native bone. Normally, low fusion rates were reported when TCP alone was used due to the only osteoconductive property without osteoinductive potential. Therefore, TCP is often combined with autologous bone graft or bone marrow aspiration (BMA) to improve osteoinductive and osteogenic properties [10, 11].

In Thailand, rhBMP-2 with an absorbable collagen sponge (Infuse^®^, Metronic Inc., Memphis, TN, USA Infuse) was introduced for use in spinal fusion as in the USA and Europe for anterior interbody fusion of L2-S1 as a substitute for autologous bone graft in adults. However, rhBMP-2 provides more clinical benefit at an incremental financial cost. Infuse^®^ kit price is 4,230 US dollar (2.8 cc of rhBMP-2) in Thailand. Recently, Parker et al. suggested that β-TCP was a viable alternative to rhBMP-2 at a lower price (1,200 US dollars per 10 cc strips of TCP) with comparable clinical outcomes and complication rates [12]. With regard to health economics, it is important to define clear criteria for the cost-effectiveness and the cost-benefit of a specific treatment material. A better outcome and cheaper cost of using one treatment will make it easier to make a decision to use those materials. Thailand is a developing upper-middle-income country, despite its excellent fusion rate and clinical results, there are limitations in using rhBMP-2. Therefore, the aim of this work was to determine clinical outcomes and the economic impact of using TCP combined with IBG compared to rhBMP-2 in minimally invasive lateral lumbar spine surgery in Thailand.

## Methods

### Patients

This retrospective study was carried out collecting data from electronic medical records and the Siriraj Spine Registry database of Siriraj Hospital, Mahidol University, Bangkok, Thailand. This study was reviewed and approved by the Siriraj Institutional Review Board (SIRB) (COA no. Si 089/2022). The requirement to obtain written informed consent was waived due to the retrospective and anonymous nature of the study.

This retrospective study included consecutive patients who underwent XLIF surgery in the Department of Orthopaedic Surgery of Siriraj Hospital, Faculty of Medicine, Bangkok, Thailand, during the 2014–2020 study period. Patients with degenerative lumbar spondylolisthesis or lumbar spinal stenosis or degenerative disc disease who were treated with XLIF surgery and who had complete clinical data and radiography of the lumbar spine at preoperative and 2-year follow-up were included. Each included patient underwent single-level lumbar interbody fusion by lateral approach between L2-5 (XLIF^®^; NuVasive, Inc., San Diego, CA, USA) with supplemental posterior instrumentation (percutaneous pedicle screw fixation). Each patient made decision on either using rhBMP-2 or β-TCP combined with IBG by themselves. A single orthopedic surgeon (WS) operated on all patients. After enrollment, each patient’s medical record and demographic data, surgical notes, anesthesia records, discharge summaries, clinical progression notes and direct medical charges were collected and assessed. Patients were excluded if (1) they had incomplete medical or surgical details or (2) could not respond to questions effectively.

The sample size calculation was based on the data of the fusion rates reported by Parker et al. of calcium phosphate bone substitute and recombinant human bone morphogenetic protein-2 for XLIF surgery at 2 years follow up [12]. The fusion rates of 25 patients who used calcium phosphate and 107 patients who used rhBMP-2, measured by radiographic evaluation, were 80% and 96%, respectively. An online statistical sample size calculator program “Statulator” was used with a power of 80% and a level of significance of 5% with non-inferiority margin 15%. The study would require a sample size of 24 for each group [13].

### Surgical techniques

Under general anesthesia, the patient was placed in the left lateral decubitus on a radiolucent table. Anteroposterior and lateral radiographic views were arranged by the surgeon using intraoperative fluoroscopy until excellent images of the lumbar spine were achieved. The patient was then prepped and draped in the routine sterile manner. The standard XLIF procedure, previously described, was performed using the mini-open lateral approach [14]. Mini-open lateral approach was started with 3-cm oblique skin incision. Then the external oblique muscle, internal oblique muscle, and transversalis muscle were dissected by using electrical cauterization, the retroperitoneal approach, and psoas muscle splitting technique were used. An insulated dilators (NVM5^®^ dilators, NuVasive, Inc, San Diego, CA, USA) combined with neuromonitoring system (NVM5^®^, NuVasive, Inc, San Diego, CA, USA) was used to confirm that it was not contiguous to lumbar plexus. After that, tubular retractor (MaXcess^®^, NuVasive, Inc, San Diego, CA, USA) was inserted. Then, discectomy with endplate preparation was performed after insertion of the interbody cage using a 10-degree lordotic intervertebral polyetheretherketone (PEEK) cage (CoRoent^®^; NuVasive, Inc, San Diego, CA, USA). The interbody PEEK cages were filled with rhBMP-2 (Infuse^®^, Metronic Inc., Memphis, TN, USA) or β-Tricalcium phosphate (AttraX^®^; NuVasive, Inc., USA) combined with Iliac crest bone graft (ICBG). After closing the wound in the the lateral decubitus position, the patient was subsequently positioned in the prone position for posterior instrumentation by percutaneous fixation of the pedicle screw (either Apollo System; Orthopeasia Co., Ltd., Samut Prakan, Thailand or Spherx PPS system; NuVasive Inc., San Diego, CA, USA). All patients were able to walk postoperatively and were discharged according to the standard protocol after spine surgery.

### Bone substitute materials

For single level XLIF surgery in this study, the interbody cages were filled with either rhBMP-2 or β-TCP combined with IBG and wrapped by absorbable suture material for preventing materials slip off. In the BMP-2 group, a small size of rhBMP-2 with an amount of 2.8 cc was applied to an absorbable collagen sponge (ACS) carrier. The carrier was cut to an appropriate size to fix a hole in the interbody cages. In the TCP + IBG group, approximately 2–4 cc of TCP combined with 1–2 cc amount of cancellous iliac bone graft that was harvested from iliac crest via mini-open technique during the lateral decubitus position was used to fill the interbody cage.

### Study procedure

The patients were divided into two groups: a “TCP + IBG group” and a “BMP-2 group”. Data related to the preoperative, perioperative, and 2-year postoperative periods were reviewed. The estimated blood loss, operative time, and length of hospital stay were used to evaluate perioperative outcomes. Patient quality of life and functional outcomes were evaluated using the EuroQol − 5 Dimensions − 5 Levels (EQ-5D-5L), the EuroQol Visual Analog Scale (EQ-VAS), and the Oswestry Disability Index. The EQ-5D-5L scores were converted to utility scores using previously published coefficient factors specific to the Thai population [15].

### Radiographic measurements

Computerized topography (CT) of the lumbar spine was collected after two years of follow-up for evaluation by two spine surgeons at our center. All radiographic evaluations were measured using an image viewer computer system (Sectra IDS7 version 15.1.28.6; Sectra AB, Linkoping, Sweden). Successful bony fusion was defined as the presence of bone bridging interbody trabecular bone in the coronal and sagittal views of CT [16–18]. The subsidence of the PEEK was determined on CT and was defined as the breaches of the adjacent endplates breaches > 2 mm [19].

### Economic measurement

Direct medical charges were obtained from the retrospective review of the data. All charges were converted to 2022 USD using the consumer price index (CPI) and the exchange rate at 1 USD = 31.98 Thai baht [20, 21]. Utility data were prospectively collected at the perioperative time points.

Direct medical expenses included all costs associated with the operation and basic postoperative hospitalization. In this category, charges were further itemized and grouped into ten categories: bone graft, instrumentation, operating room services, anesthesia, surgical supplies, transfusion, room and board, medications, laboratory, and physical and occupational therapy.

### Statistical analysis

The demographic and clinical characteristics of the patients were analyzed and descriptively reported. Categorical data were compared using the chi-square test and Fisher’s exact test, as appropriate. The distribution of continuous numeric data was checked by using Shapiro-wilk test. Normally distributed continuous data were compared using an independent t-test. The results are presented as mean ± standard deviation and frequency (percentage). If the data was not normally distributed, non-parametric test such as Mann-Whitney U test or Wilcoxon signed-rank test was using for calculation. Median (Inter-quartile range; IQR) was used for representing non-parametric outcome parameters. A 2-tailed probability (P) value of less than 0.05 was deemed statistically significant. All data analyses were carried out with IBM SPSS Statistics for Windows, version 19.0 (IBM Corp, Armonk, NY, USA).

## Result

In total, 55 consecutive patients were identified as having undergone single-level lumbar fusion with the XLIF procedure in our department between 2014 and 2020 and were followed for 2 years. Of these, 25 patients used TCP combined with IBG (TCP + IBG) and 30 patients used BMP-2. Demographic data from both groups are shown in Table 1. The mean age of the TCP + IBG and BMP-2 groups was not statistically different at 60.08 ± 11.03 years and 64.53 ± 11.99 years, respectively (P = 0.16). There were no statistically significant differences in sex (P = 0.87), underlying diseases (P = 0.28), diagnosis (P = 0.11), and spine level (P = 0.23). Most of the patients in both groups were women (72% in TCP + IBG and 70% in BMP-2) and hypertension was the most common underlying disease. Spinal stenosis was the most common diagnosis for the TCP + IBG group (48%), while spondylolisthesis was the most common primary diagnosis for the BMP-2 group (60%). L4-5 were the most common surgical levels in both groups (TCP + IBG: 80% and BMP-2: 66.7%).

**Table 1:** Demographic data

Characteristics	TCP + IBG (n = 25)	BMP-2 (n = 30)	P value
**Gender**, n (%)			
Male	7(28)	9(30)	0.87
Female	18(72)	21(70)	
**Age (years)** (mean ± SD)	60.08 ± 11.03	64.53 ± 11.99	0.16
**Underlying diseases**, n (%)			
Hypertension	14(56)	16(53.3)	
DM	4(16)	4(13.3)	0.28
Coronary artery disease	0(0)	3(10)	
**Diagnosis**, n (%)			
Spinal stenosis	12(48)	11(36.7)	
Spondylolisthesis	9(36)	18(60)	0.11
Degenerative disc disease	4(16)	1(3.3)	
**Level of Spine**, n (%)			
L2–L3	2(8)	1(3.3)	
L3–L4	3(12)	9(30)	0.23
L4–L5	20(80)	20(66.7)	

*Significant at a P value of less than 0.05. Statistical analysis by unpaired Student’s t-test, the chi-square test and Fisher’s exact test. TCP + IBG: Tricalcium phosphate plus iliac bone graft, BMP-2: Bone Morphologic Protein-2, DM: Diabetes Mellitus

### Perioperative parameters

The TCP + IBG group had a larger median estimate of blood loss compared to the BMP-2 group (200 (100,. 250) ml of TCP + IBG vs. 100 (50, 162.5) ml of BMP-2; P = 0.02). However, there were no statistically significant differences in median operative time (TCP + IBG: 205 (170, 292.5) min vs. BMP-2: 187.5 (155, 255) min, P = 0.14) and in median hospital stay (TCP + IBG: 6 (5, 9) days vs. BMP-2: 6 (5, 8) days, P = 0.43).

There were no serious complications in our study during the follow-up period. Complications were significantly more frequent in the TCP + IBG group compared to the BMP-2 group during the perioperative period (24% vs. 3.3%, respectively, P = 0.04) as shown in Table 2. Postoperative anemia occurred in four patients in the TCP + IBG group and in one patient in the BMP-2 group. For proximal limb neuropathy, there was no statistically significant difference in TCP + IBG and BMP-2 and (36.4% and 63.6%, respectively) (P = 0.41). There were no late complications related to BMP-2 such as ectopic bone formation or radiculopathy or tumor formation during 2-year postoperative follow up. The fusion rate seemed to be higher in BMP-2 group but did not differ significantly at two years of postoperative follow-up (P = 0.08). The TCP + IBG group demonstrated fusion in 20/25 (80%), and the BMP-2 group resulted in fusion in 29/30 (96.7%). In addition, there was no significant difference in the PEEK subsidence rate in both groups (TCP + IBG: 44% vs. BMP-2: 23.3%, P = 0.15).

**Table 2:** Perioperative data, postoperative complications, fusion rate, and PEEK subsidence rate

Characteristics	TCP + IBG (n = 25)	BMP-2 (n = 30)	P value
**Estimate blood loss (ml)** (median (IQR))	200 (100, 250)	100 (50, 162.5)	0.02*
**Operative time (min)** (median (IQR))	205 (170, 292.5)	187.5 (155, 255)	0.14
**Length of hospital stay (days)** (Median (IQR))	6 (5, 9)	6 (5, 8)	0.43
**Post operative complications**, n (%)	6/25 (24)	1/30 (3.3)	0.04*
Hematoma Electrolyte imbalance	1 2	0 0	
Anemia	4	1	
**Proximal limb neuropathy**, n(%) **Fusion rate**, n(%)	8/25 (36.4) 20/25 (80)	14/30 (63.6) 29/30 (96.7)	0.41 0.08
**PEEK subsidence rate**, n(%)	11/25 (44.0)	7/30 (23.3)	0.15

*Significant at a P value of less than 0.05. Statistical analysis by Mann-Whitney U test and Fisher’s exact test. TCP + IBG: Tricalcium phosphate plus iliac bone graft, BMP-2: Bone Morphologic Protein-2, PEEK: Polyetheretherketone

### Clinical and health-related quality of life outcomes

The clinical results at the preoperative and 2-year postoperative follow-up are shown in Table 3. At the preoperative stage, there were no statistically significant differences in utility (P = 0.36), ODI (P = 0.44) and EQ-VAS (P = 0.77) between the TCP + IBG group and the BMP-2 group. All clinical outcomes (utility, ODI and EQ-VAS) improved significantly in both groups compared to the preoperative and 2-year follow up (P < 0.05). However, there were no differences at the 2-year postoperative follow-up in utility (P = 0.55), ODI (P = 0.43) and EQ-VAS (P = 0.82) between the TCP + IBG group and the BMP-2 group.

**Table 3:** Utility, ODI, and EQ-VAS of BMP-2 and TCP + IBG preoperatively and at the 2-year follow up

Parameters	TCP + IBG (n = 25)		P value	BMP-2 (n = 30)		P value	2-year compare
	Preop	2-year		Preop	2-year		
Utility	0.71 ± 0.21	0.86 ± 0.13	< 0.001*	0.65 ± 0.25	0.83 ± 0.17	< 0.001*	0.55
ODI	38.26 ± 24.05	23.57 ± 17.31	0.005*	34.07 ± 16.52	20.16 ± 14.66	< 0.001*	0.43
EQ-VAS	65.2 ± 16.61	77.0 ± 18.76	0.024*	63.63 ± 21.64	75.83 ± 18.89	0.006*	0.82

*Significant at a p-value of less than 0.05. Statistical analysis by Student’s t-test both unpaired and paired t-test. TCP + IBG: Tricalcium phosphate plus iliac bone graft, BMP-2: Bone Morphologic Protein-2, ODI: Oswestry Disability Index, EQ-VAS: EuroQol-Visual Analog Scale

### Economic outcomes

**Fig. 1:**
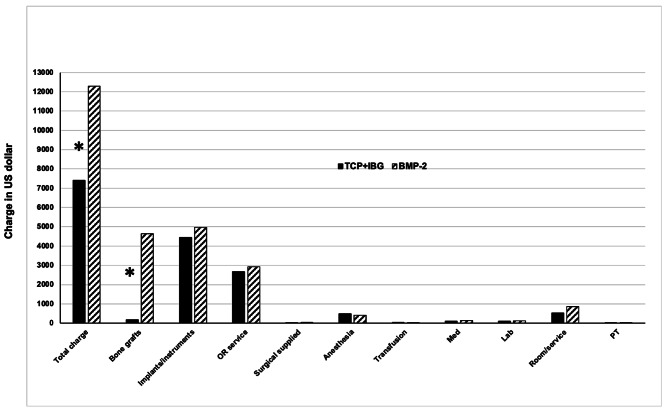
Average means of TCP + IBG and BMP-2 original procedure costs *Significance p-value less than 0.05. Statistical analysis by student unpaired t-test TCP + IBG: Tricalcium phosphate plus iliac bone graft, BMP-2: Bone Morphologic Protein-2, OR: Operative room, Med: Medication, Lab: Laboratory, PT: Physical therapy

The average total charges for TCP + IBG and BMP-2 were 7,413.1 ± 1,683.9 USD and 12,296.7 ± 3,164.7 USD, respectively, for the perioperative time with statistically significant difference (P < 0.001). The average savings for TCP + IBG over BMP-2 was 4,883.6 USD or 39.7% of the total charge per patient. Data for the detail charge categories (bone graft, implants / instruments, ambulatory service, surgically supplied, anesthesia, transfusion, medication, laboratory, room/service, and physical therapy) are shown in Fig. 1. The implants / instruments charges were 4,436.9 ± 1264.4 USD for TCP + IBG and 4,974.7 ± 860 USD for BMP-2, which represented 59.8% and 40.4% of the total charge without statistical difference (P = 0.06). Comparison of bone graft charges between TCP + IBG (179.2 ± 50.9 USD) and BMP-2 (4,632.2 ± 543.1 USD) showed BMP-2 charges that exceeded TCP + IBG by 4,453 USD with statistically significant (P < 0.001). However, other categories of charges (OR service, surgical supplies, anesthesia, transfusion, medication, laboratory, room/service, and physical therapy) were not significantly different (P > 0.05) between TCP + IBG and BMP-2 (P > 0.05).

## Discussion

Minimally invasive lateral interbody fusion, especially XLIF, has gained popularity in the treatment of degenerative spine disease due to excellent results with less damage to the paraspinal muscles and ligaments [14]. Many studies demonstrated a significant improvement in clinical and radiographic outcomes following XLIF surgery [22]. Most studies on XLIF use rhBMP-2 as a bone graft replacement; only a few studies have used other bone graft materials such as calcium phosphate [12, 23, 24]. In addition, none of the previous studies used calcium phosphate combined with iliac bone graft in XLIF surgery. To our knowledge, our study was the first to evaluate clinical, radiographic, and economic outcomes compared between the use of tricalcium phosphate combined with an iliac bone graft and rhBMP-2.

We compared the clinical, radiographic, and economic outcomes of 25 patients with TCP + IBG with 30 patients with BMP-2. There were no significant differences between these two groups in demographic data. During the perioperative period, the mean estimate of blood loss and postoperative complications were significantly higher in TCP + IBG. Operative time, length of hospital stay, and proximal limb neuropathy were equally in these two groups. On radiographic evaluation at 2 years of follow-up, there was a lower fusion rate and a higher PEEK subsidence rate in the TCP + IBG group than in the BMP-2 group, but there was no statistically significant difference. In addition to equally in radiographic evaluation, our group also did not demonstrate significant differences in any clinical outcome (Utility, ODI, and EQ-VAS) between TCP + IBG and BMP-2 patients. In Thailand, the total cost for 1-level XLIF surgery (12,296.7 USD) is lower compared to the USA (24,320.16 USD) [25]. Most of the charge in XLIF surgery was based on the charge of the bone graft and instrumentation. Our group was able to reduce both total charges and bone graft charges by up to 50% when TCP + IBG was used as bone graft replacement.

In our study, there were no significant differences in radiographic and clinical outcomes between the TCP + IBG group and the BMP-2 group after 2-year follow-up, which corresponds to studies by Parker et al. [12] and of Pimenta et al. [24]. Therefore, our study also supported that tricalcium phosphate combined with the iliac bone graft can be used as a replacement for the bone graft in XLIF surgery with posterior instrumentation with a radiographic and clinical outcome similar to rhBMP-2.

The fusion rate of the BMP-2 group in our study (29/30; 96.7%) is equal to the fusion rate in the instrumentation surgery group in the previous study by Parker et al. (67/67; 100%). However, the fusion rate of the TCP + IBG group in our study (20/25; 80%) was lower compared to the TCP group with instrumentation in the study by Parker et al. (10/11; 91%). Furthermore, a recent prospective randomized single-center study by Menezes et al. demonstrated a higher fusion rate (27/28; 96.4%) when using tricalcium phosphate as a stand-alone bone graft substitute for single-level and single-position XLIF surgery.

The increased blood loss that we observed could be explained by bleeding during the iliac bone graft harvesting procedure. This also led to postoperative hematoma at the harvest site and anemia in the TCP + IBG group. The hematoma of all patients resolved within one month of follow-up.

The prevalence of proximal limb neuropathy after XLIF surgery in this study was 22/55 (40%), which was equivalent to a previous study of between 19% and 67% [26, 27]. There were no differences in these complications between these two groups. At 3 months of follow-up, this proximal limb neuropathy resolved spontaneously in up to 75% of these patients.

Our economic analysis demonstrated the lower total charge of XLIF surgery in Thailand (TCP + IBG: 7,413.1 ± 1,683.9 USD and BMP-2: 12,296.7 ± 3,164.7 USD) when compared to those in the USA (24,320.16 USD in the Lucio et al. study and 20,515.77 USD in the Hartman et al. study) [25, 28]. The mean charge of the bone graft in Thailand (TCP: 179.2 USD and BMP-2: 4,632.2 USD) is equivalent to the cost in USA. The lower total charges in Thailand can be explained by the lower charges of the other nine categories (implants/instrumentation, OR service, surgical supplies, anesthesia, transfusion, medication, laboratory, room/service, and physical therapy) in Thailand compared to those in the United States [25]. The mean instrumentation charges in Thailand (BMP-2: 4,974.7 USD and TCP + IBG: 4,436.9 USD) are less than the instrumentation charges in the USA by the study Hartmann et al. (6,716 USD) [28]. To our knowledge, our study was the first to demonstrate a significantly lower total charge in TCP + IBG than in BMP-2 (approximately 39.7%). In the Parker et al. study only the cost of bone grafts was described, which showed a lower cost of bone grafts in TCP, even when instrumentation costs were included [12]. Therefore, it may be more cost-effective for the XLIF patient using TCP + IBG in Thailand in terms of cost savings on surgery and obtain radiographic and clinical outcomes similar to those of rhBMP-2.

This study has some limitations. This study was a retrospective, nonrandomized single center study that may have introduced selection bias. We need further studies with a more valid method, such as cost-effectiveness and cost-utility analyses, to investigate and confirm the results of treatments in terms of their cost-effectiveness in the short and long term. In this study, we did not investigate the actual costs of the treatments due to limited data. However, the cost-to-charge ratio of Siriraj Hospital was approximately 1, and we could infer that our results could also represent the costs of treatments from a healthcare provider’s perspective. In addition, a low number of patients in each category may affect statistical analysis and result interpretation. The strengths of this study include the calculation of the sample size, the 2-year follow-up, and the use of two independent orthopedic surgeons in the evaluation of the radiographic data.

## Conclusion

The use of TCP + IBG as a standalone bone substitution for XLIF surgery with additional posterior instrumentation results in significant reductions in direct medical charges compared to those using rhBMP-2 in the perioperative period. Additionally, patients with TCP + IBG have long-term outcomes similar to those of radiographic and clinical outcomes as patients with rhBMP-2. We conclude that TCP + IBG is a valuable alternative bone graft approach, especially in countries with limited resources.

## Data Availability

The datasets used and/or analyzed during the current study are available from the corresponding authors on reasonable request.
